# Profiling intraoral neuropathic disturbances following lingual nerve injury and in burning mouth syndrome

**DOI:** 10.1186/s12903-017-0360-y

**Published:** 2017-03-23

**Authors:** Amely Hartmann, Robin Seeberger, Malte Bittner, Roman Rolke, Claudia Welte-Jzyk, Monika Daubländer

**Affiliations:** 1 Private Practice Dr. Seiler und Kollegen, Echterdinger Str. 7, 70794 Filderstadt, Germany; 2 Private Practice MKG Solitude, Ludwigsburg, Germany; 3 Private Practice Dr. Meschenmoser und Dr. Bittner, Stuttgart, Germany; 4 Department of Palliative Medicine, University of Aachen, Aachen, Germany; 5 Department of Oral Surgery, University Medical Centre of the Johannes Gutenberg University of Mainz, Mainz, Germany

**Keywords:** Lingual nerve impairment, Quantitative Sensory Testing, Burning mouth syndrome, Neurosensory disturbances

## Abstract

**Background:**

The aim of the study was to analyse intraoral neurophysiological changes in patients with unilateral lingual nerve lesions as well as patients with Burning Mouth Syndrome (BMS) by applying a standardized Quantitative Sensory Testing (QST) protocol.

**Methods:**

The study included patients suffering from a peripheral lesion of the lingual nerve (*n* = 4), from BMS (*n* = 5) and healthy controls (*n* = 8). Neurophysiological tests were performed in the innervation areas of the tongue bilaterally. For BMS patients the dorsal foot area was used as reference.

**Results:**

For patients with peripheral lesion of the lingual nerve the affected side of the tongue showed increased thresholds for thermal (*p* < 0.05–0.001) and mechanical (*p* < 0.01–0.001) QST parameters, indicating a hypoesthesia and thermal hypofunction. In BMS patients, a pinprick hypoalgesia (*p* < 0.001), a cold hyperalgesia (*p* < 0.01) and cold/warmth hypoesthesia (*p* < 0.01) could be detected.

**Conclusions:**

The results of this study verified the lingual nerve lesion in our patients as a peripheral dysfunction. The profile showed a loss of sensory function for small and large fibre mediated stimuli. A more differentiated classification of the lingual nerve injury was possible with QST, regarding profile, type and severity of the neurologic lesion. BMS could be seen as neuropathy with variable central and peripheral contributions among individuals resulting in chronic pain.

## Background

Temporary or permanent inferior alveolar nerve injury (IANI) or lingual nerve impairment (LNI) are well-recognized complications of oral and maxillofacial surgery procedures [1–5]. A loss of sensory function in lower lip, chin and tongue may be a result, which could compromise talking, drinking and eating [6]. LNI may cause speech problems [7, 8].

Trigeminal nerve injury is complex [9]. Most nerves with axonal damage recover incompletely one year after damage due to surgery [10]. Ischemia or compression may cause demyelisation of the nerve impairing signal conduction in the affected nerve fiber [11]. Previous studies proved that trigeminal nerve damage may also lead to chronic neuropathic pain syndromes [12–15]. In the field of oral surgery, IANI and LNI consecutively cause litigation and patient complaints [16]. Underlying pathophysiologic mechanisms are still under investigation and sufficient diagnostic and treatment modalities are needed urgently [9]. Third molar removal caused 73% of all documented LNI’s [8]. LNI can be due to direct needle trauma to the fascicles caused by local anesthetic injection (17%) or causing hemorrhage within the epineurium or a neurotoxic effect of the anesthetic agens [9, 17, 18]. Beside this, a possible reason for injury to the lingual nerve is slitting of the submandibular duct, which was reported in 2.4% of all cases [19].

IANI was caused by more diverse procedures including third molar removal (60%), local anesthesia (19%), implants (18%) and endodontics (8%) [8].

Burning mouth syndrome (BMS) is an intraoral chronic pain condition with an intra-oral burning sensation of the mucosa [20–22]. According to the International Headache Society, BMS is defined as “an intra-oral burning or dysesthetic sensation, recurring daily for more than 2 h for more than 3 months, without clinically evident causative lesions” [23]. The International Association for the Study of Pain described BMS as “burning pain of the tongue and/or other oral mucous membrane in the absence of clinical signs or laboratory findings” [24]. Currently, the prevalence of BMS in the general population is estimated to be 0.7 to 15% [25]. BMS occurs without clinical mucosal abnormalities or lesions and without any other local or systemic reasons.

Both pathogenic profiles still represent challenges in daily practice and the underlying neuropathic mechanism is not fully understood yet.

The aim of this prospective clinical study was to profile the neurophysiological changes according to lingual nerve injury after oral surgery treatment and BMS in order to present a sufficient screening technique to objectify intraoral neurological deficiencies. A comparison of neurophysiological pathways should be derived.

## Methods

This prospective clinical monocenter study was in accordance with the 1964 Declaration of Helsinki on medical protocol and ethics. Ethical approval was permitted by the local ethics committee of Rhineland-Palatinate (837.168.13 (8853)). Informed consent of all patients and volunteers was mandatory.

### Study population

All patients were recruited by the same trained examiner (MD). BMS patients were recruited in accordance with the IHS criteria for BMS [25, 26]. All of them had a burning sensation over the tongue. Systemic diseases as well as local irritations were excluded previous to measurements. LNI patients were included presenting lingual nerve impairment after maxillofacial and dental surgery more than one year ago. Reasons for impairment were local anaesthetic injection, multiple surgery after recurrence of a peripheral giant cell granuloma, tumour surgery and other dental treatment. Diagnosis was set in accordance with the IHS.

#### Healthy volunteers

Oral sensory functions were evaluated by psychophysical means in eight healthy feminine volunteers (age range between 37 and 69 years, mean 56.9 years ±13,02). Exclusion criteria were previous orofacial injuries, diabetes, neurological and psychiatric history as well as medication within 48 h. Two volunteers took regular pain medication against headache. They were advised to stop medication 48 h prior to QST.

These healthy volunteers were compared to group A and B.

#### Group A

Four feminine patients with peripheral injury of the lingual nerve were included in the study group A (range of age 39 to 72 years; mean 50.5 years ±14,9). Patients underwent standardized testing procedures and were examined by one investigator (RS). Three patients suffered from a trauma of the LN on the right side, one patient showed nerve impairment on the left side. One patient suffering from trauma on the right side took pain killers and anticonvulsants regularly. She was advised to stop medication 48 h prior to investigation. QST measurements concerned the innervation areas of the affected nerves and interindividually compared with the contralateral unaffected side. Patients had to specify the type of sensory impairment.

#### Group B

Five patients (4 women, 1 man in the range of age 37 to 70 years; mean 51.8 years ± 12,9) with BMS were analysed by standardized testing procedures and were examined by one investigator (MB). The timespan of discomfort ranged from 14 to 84 months. Two of them took pain killers and anticonvulsants regularly. They stopped medication 48 h before measurements. As the whole tongue is affected, QST measurements were carried out in the anterior part of the tongue and compared with healthy volunteers. Additionally, measurements were performed on the dorsal foot area in each patient to preclude general sensitisation. Patients had to specify the type of sensory impairment.

### Method

The QST protocol used in this study based on the comprehensive protocol of the German Research Network on Neuropathic Pain (DFNS) as described by Rolke et al. [27] and consisted of the following parameters:

CDT (cold detection threshold); WDT (warm detection threshold); TSL (thermal sensory limen); PHS (paradoxical heat sensation); CPT (cold pain threshold); HPT (heat pain threshold); MDT (mechanical detection threshold); MPT (mechanical pain threshold); MPS (mechanical pain sensitivity); DMA (dynamic mechanical allodynia); WUR (wind up ratio); VDT (vibration detection threshold) and PPT (pressure pain threshold).

QST was performed in all patients and healthy controls at the Department of Oral Surgery, University Medical Centre of the Johannes Gutenberg University of Mainz, Germany. Measurements for one patient took about 90 min. In both patient groups, QST evaluated the left and the right side of the tongue. To standardize the testing procedure, tests concerned the anterior third lateral side of the tongue. In patients with BMS, we used the dorsal area of the foot as control side. Mechanical and thermal tests were performed according to our previous study [28]:

#### Thermal tests

CDT, TSL, WDT, CPT and HPT were determined by using a computer-controlled Peltier – type thermode (stimulation area 9*9 cm^2^, Somedic® Sales AB, Hörby, Sweden) and the Classic Method of Limits [29, 30]. Baseline temperature was set to 32 °C and temperature modulation was performed between 5 and 50 °C. CDT and WDT represent temperature difference from 32 °C non-noxious to warm or cold. The first painful cold (CPT) or hot (HPT) sensation was measured and TSL was determined by alternating warm and cold stimuli. Possible PHS was requested as a subjective feeling of heat upon cooling.

The investigators instructed the patients to press a button as soon as they noticed the required sensation. Patients and healthy volunteers had to keep the eyes closed during the whole QST procedure. Three repetitive measurements reduced bias and thresholds were marked as mean thresholds.

#### Mechanical tests

MDT was investigated with a standardized set of modified von Frey filaments (Optihair2®-Set, Marstock Nervtest, Germany) and using the “Method of Limits” with forces between 0.25 and 512 mN graded by a factor of 2 (1–2 s contact time). The contact area of the von Frey filaments with the tongue was of uniform size and shape (rounded tip, 0.5 mm in diameter).

For MPT, custom-made weighted pinprick stimuli with a flat contact area of 0.25 diameter were used. Fixed stimulus intensities with forces of 8, 16, 32, 64, 128, 256 and 512 mN were applied at a rate of 2 s on, 2 s off again in a series of ascending and descending force intensities. Cut-off was the first sensation of sharpness appeared or disappeared. Five repetitive measurements were performed for MDT and MPT and calculation meant the geometric mean of these 5 series.

Rating pain intensity was evaluated for each stimulus on a ‘0–100’ scale (‘0’ indicating “no pain” and ‘100’ indicating “maximum imaginable pain”), MPS was measured using pinprick stimuli and calculated as the geometric mean of all numerical ratings. Soft stimuli with a cotton wisp exerting a force of ~5 mN, a cotton wool tip fixed to an elastic strip (~100 mN) and a cotton wool tip fixed to a stiff strip (~200–400 mN) indicated DMA. Stimuli were applied with a single stroke of approximately 1 cm in length over the tongue at each site and inserted into the protocol of the pinprick stimuli. Patients and healthy volunteers gave numerical pain ratings for each stimulus, given in runs of 10 in pseudorandomized sequence in a ~10 s inter-stimulus interval. Allodynia was calculated as the geometric mean of numerical ratings across the three different types of light touch stimulators.

WUR was evaluated by applying repeated painful stimuli (single pinprick stimulus 128mN compared to a series of 10 repetitive pinprick stimuli of same intensity).

The subject was asked to give a pain rating representing the single stimulus and the estimated mean over the whole series of 10 stimuli using a ‘0–100’ numerical rating scale. The whole procedure was repeated five times. WUR was calculated as the ratio: mean rating of the five series divided by the mean rating of the five single stimuli. VDT was investigated by applying a Rydel–Seiffer (Aesculap, Tuttlingen, Germany) graded tuning fork (64 Hz, 8/8 scale) over the tongue in three stimulus repetitions. Individuals had to notice disappearance of the vibration sensation. Within three series of ascending stimulus intensities, PPT was measured as the first painful sensation. The applied pressure gauge device with a probe diameter of 1.1 cm, FDN200, Wagner Instruments, USA) exerted forces up to 20 kg/cm^2^ (~2000 kPa) with an increasing ramp of 50 kPa/s per series (0.5 kg/cm^2^ per second).

### Data evaluation

Data evaluation was performed as described in the standardized protocol of the German Research Network on Neuropathic Pain [27, 31]. All statistical calculations were measured using Statistica® software for Windows (StatSoft® Inc., USA) and presented as mean ± standard deviation (SD).

For comparison of differences between Z-score QST data (patient groups/ controls) two-way analysis of variance (ANOVA) was used. LSD-post hoc tests (LSD; least significant difference) was performed to evaluate post hoc comparisons. In case of missing dynamic mechanical allodynia (DMA) healthy controls, raw data were tested using unpaired *t*-test in comparison to the expected value of zero.

Results of the control group were used to normalize test results visualized by calculating the z-transform: Z = (value (patient) – mean (controls)) / SD (controls). To compare reference data between left and right, QST data were estimated by subtraction of the left-tongue side from the right-tongue side respectively. In case of an estimated mean right–left zero value, the 95% confidence interval of relative reference data was calculated as zero ±1.96 SD.

## Results

### Demographics

QST protocol was used for assessment of sensory parameters in subjects with LNI (*n* = 4, Group A), BMS (*n* = 5, Group B) and controls (*n* = 8). 8 (89%) patients were female and compared to 8 healthy female volunteers with a mean age of 56.9 years ±13,02 (age range between 37 and 69 years). Study group A consisted of 4 women with a mean age of 50.5 years ±14,9 (range of age 39 to 72 years). Study group B consisted of 4 women and 1 man with a mean age of 51.8 years ±12,9 (range of age 37 to 70 years).

The mean duration of symptoms experienced by the LNI group was longer at 58.5 months compared with the BMS patients who had to cope with their symptoms for a mean duration of 22.8 months. The timespan of discomfort in LNI group ranged from 24 to 102 months and in BMS group from 12 to 42 months.

### QST measurements

**Fig. 1:**
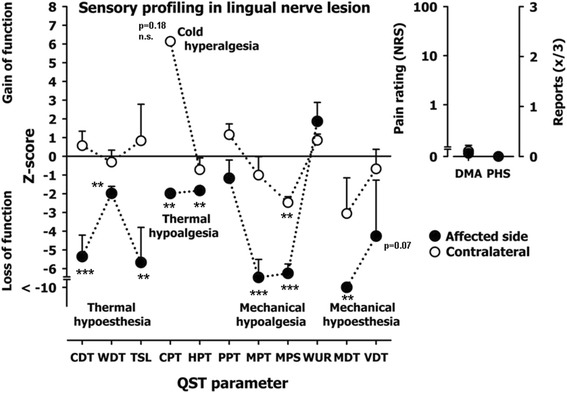
Z-score QST profiles of the tongue in patients with LNI. A Z-score of 0 means the score is the same as for the mean of the healthy subjects. It can also be negative or positive indicating loss or gain of function. Affected side (filled-circles) represent the QST profiles of all patient’s affected tongue sides suffering from a peripheral lesion of the lingual nerve. The profile shows a combined loss of sensory function for small fiber mediated stimuli and for large fiber mediated stimuli (note the mechanical detection threshold for von Frey-filaments (MDT), and the vibration detection threshold (VDT). Contralateral (open circles) show the QST profile of all patient’s healthy sides of the tongue. Z-Score range represents physiologic values of healthy volunteers. *p < 0.05; **p < 0.01, ***p < 0.001; n.s. = no significance

- LNI patients affected side compared to healthy controls (group difference) (Table 1 and 2, Fig. 1)

**Table 1:** Quantitative sensory testing results in patients with lingual nerve impairment (LNI) - Data presented patients’ affected and non-affected sides as well as controls’ left and right side of the tongue

QST parameter				
	Log-transformed data^a^			
	Patients (tongue sides)		Controls (tongue side)	
	Affected	Contralateral	Right	Left
CDT (°C)	1.2 ± 0.3	0.5 ± 0.2	0.6 ± 0.1	0.6 ± 0.1
WDT (°C)	1.2 ± 0.2	0.8 ± 0.3	0.7 ± 0.2	0.7 ± 0.2
TSL (°C)	1.5 ± 0.2	1.1 ± 0.2	1.1 ± 0.1	1.1 ± 0.1
CPT (°C)				
HPT (°C)				
PPT (kPa)	2.3 ± 0.2	2.0 ± 0.1	2.2 ± 0.1	2.2 ± 0.1
MPT (mN)	2.5 ± 0.3	1.7 ± 0.3	1.5 ± 0.2	1.5 ± 0.2
MPS (rating 0–100)	−0.6 ± 0.2	0.04 ± 0.1	0.4 ± 0.2	0.4 ± 0.2
WUR (ratio)	0.5 ± 0.4	0.3 ± 0.1	0.2 ± 0.2	0.2 ± 0.2
MDT (mN)	0.7 ± 1.2	−0.3 ± 0.4	−0.7 ± 0.1	−0.6 ± 0.1
VDT (x/8)				
	Data in original units^a, b^			
	Patients (tongue sides)		Controls (tongue side)	
	Affected	Contralateral	Right	Left
CDT (°C)	−15.7	−3.4	−4.1	−4
WDT (°C)	14.8	6.3	5.4	5.4
TSL (°C)	29.2	12.5	14	13.9
CPT (°C)	5 (cut off) ± 0	10.5 ± 8.5	6.6 ± 0.8	6.4 ± 0.7
HPT (°C)	50 ± 0	48.4 ± 2.1	46.9 ± 1.7	47 ± 1.6
PPT (kPa)	186.2	108.3	142.8	141.4
MPT (mN)	279.2	44.5	30.7	30.9
MPS (rating 0–100)	0.2	1.1	2.6	2.6
WUR (ratio)	3.1	2.1	1.5	1.5
MDT (mN)	5.5	0.5	0.2	0.2
VDT (x/8)	3.8 ± 2.6	5.4 ± 1.1	5.6 ± 0.4	5.8 ± 0.5

^a^For CDT and WDT, differences from baseline-temperature (32 °C) are shown

^b^Retransformed mean for log-normally distributed data. All data are presented as mean ± SD. In the case of CPT, HPT and VDT, mean original data ± SD are shown. There is no evidence of occurrence of DMA and PHS

*CDT* Cold detection threshold, *WDT* Warm detection threshold, *TSL* Thermal sensory limen, *CPT* Cold pain threshold, *HPT* Heat pain threshold, *PPT* Pressure pain threshold, *MPT* Mechanical pain threshold, *MPS* Mechanical pain sensitivity, *WUR* Wind-up ratio, *MDT* Mechanical detection threshold, *VDT* Vibration detection threshold, *DMA* Dynamic mechanical allodynia, *PHS* Paradoxical heat sensation

**Table 2:** Comparison of the QST parameters in patients with lingual nerve impairment (LNI) (Results of the ANOVA – LSD posthoc analysis)

	1) Group difference			2) Side-difference			3) Interaction group/side		
	LNI versus control group			LNI versus uninjured side					
	ANOVA Factor		LSD posthoc	ANOVA Factor		LSD posthoc	ANOVA Factor		LSD posthoc
QST Parameter	F-value	P-value		F-value	P-value		F-value	P-value	
CDT	11	<0.01 **	<0.001 ***	42.2	<0.001 ***	<0.001 ***	39.3	<0.001 ***	<0.001 ***
WDT	3.9	n.s.	<0.05 *	18	<0.01 **	<0.001 ***	17.6	<0.01 **	<0.01 **
TSL	7.1	<0.05 *	<0.001 ***	11.6	<0.01 **	<0.01 *	11.2	<0.01 **	<0.001 ***
CPT	0.8	n.s.	n.s.	3.5	n.s.	<0.05 *	4	n.s.	n.s.
HPT	5.5	<0.05 *	<0.05 *	5.9	<0.05 *	<0.01 **	7.6	<0.05 *	<0.01 **
PPT	0	n.s.	n.s.	8.3	<0.05 *	<0.01 **	7.7	<0.05 *	n.s.
MPT	36.8	<0.001 ***	<0.001 ***	20.4	<0.001 ***	<0.001 ***	20.8	<0.001 ***	<0.001 ***
MPS	58.9	<0.001 ***	<0.001 ***	483.3	<0.001 ***	<0.001 ***	492.4	<0.001 ***	<0.001 ***
WUR	4.5	n.s.	n.s.	2.4	n.s.	n.s.	2.3	n.s.	<0.05 *
MDT	11.9	<0.01 **	<0.01 **	7.9	<0.05 *	<0.01 **	8.7	<0.05 *	<0.001 ***
VDT	3.2	n.s.	n.s.	6.7	<0.05 *	<0.05 *	4.5	n.s.	<0.05 *
DMA	2.8	n.s.	n.s.	0.1	n.s.	n.s.	0.3	n.s.	n.s.

1) LNI patients affected side compared to healthy controls (group difference)

2) LNI patients affected side compared to contralateral control side (side difference)

3) LNI patients affected side compared to contralateral and healthy control group (interaction group/side)

QST was performed as a split study on the left and right side of the tongue on patients (*n* = 4) and healthy controls (*n* = 8). An ANOVA as well as a LSD posthoc (groups/ side/ interaction) was calculated to indicate main effects in the comparison of the LNI and the control group (group difference), the LNI and uninjured side of the tongue (side-difference) and for the interaction among each other (interaction group/side) *p < 0.05; **p < 0.01, ***p < 0.001; n.s. = no significance, significance shown in Fig. 1

Comparing patients’ affected side of the tongue with healthy volunteers a significant difference in the perception was evaluated for several QST parameters. Compared to controls, the affected side showed a significant thermal hypoesthesia represented by CDT *p* < 0.001 WDT *p* < 0.05 and TSL *p* < 0.001 and a significant thermal hypoalgesia represented by HPT *p* < 0.05 on the side with the lesion. For mechanical parameters, significant diversity was found for MPT (*p* < 0.001), MPS (*p* < 0.001) and MDT (*p* < 0.01). No PHS occurred and no significant DMA was found. For WUR, VDT and PPT showed no significant difference compared to healthy control side.

- LNI patients affected side compared to contralateral control side (side difference) (Tables 1 and 2, Fig. 1)

Comparing the testing areas (right and left side of the tongue) almost all QST parameters showed significant differences (CDT *p* < 0.001, WDT *p* < 0.001, TSL *p* < 0.01 CPT *p* < 0.05, HPT *p* < 0.01, MDT *p* > 0.01, MPT *p* < 0.001, MPS *p* < 0.001, PPT *p* < 0.01, VDT *p* > 0.05). Exceptions were WUR and DMA. These parameters showed no significant loss of function when comparing healthy and affected side.

- LNI patients affected side compared to contralateral and healthy control group (interaction group/side) (Table 2)

Comparing affected sides with contralateral and healthy control group, significant difference comparable to inter-individual tests were measured.

**Fig. 2:**
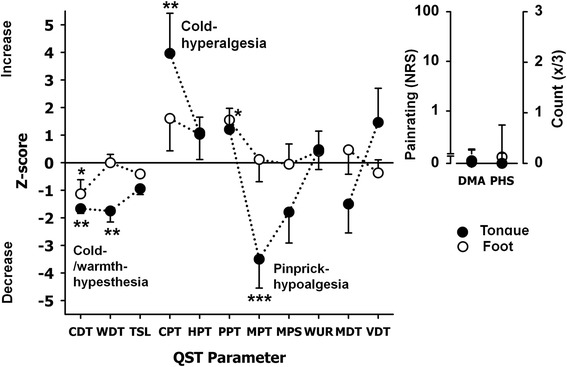
Z-score QST profiles of the tongue in patients with BMS. A Z-score of 0 means the score is the same as for the mean of the healthy subjects. It can also be negative or positive indicating loss or gain of function. Filled-circles represent the QST profiles of the tongue for all patient’s suffering from burning mouth syndrome. Open circles show the QST profile of all patient’s foot. Z-Score range represents physiologic values of healthy volunteers. The profile shows a predominant small fiber deficit; which is possible a conditioning input to central neurons and thus mediates cold hyperalgesia and also Pinprick hypoalgesia. For DMA and PHS there was no significant difference. (*p < 0.05, **p < 0.01, ***p < 0.001)

- BMS patients tongue compared to healthy controls tongue (group difference) (Tables 3 and 4, Fig. 2)

**Table 3:** Quantitative sensory testing results in patients with burning mouth syndrome (BMS) – Data presented patients affected tongue and dorsal foot as well as controls tongue and dorsal foot

QST parameter				
	Log-transformed data^a^			
	Patients		Controls	
	Tongue	Foot	Tongue	Foot
CDT (°C)	0.4 ± 0.2	0.6 ± 0.3	−0.4 ± 0.5	0.3 ± 0.2
WDT (°C	0.5 ± 0.1	0.6 ± 0.2	0.2 ± 0.2	0.6 ± 0.2
TSL (°C)	0.6 ± 0.1	0.9 ± 0.1	0.3 ± 0.3	0.8 ± 0.3
CPT (°C)				
HPT (°C)				
PPT (kPa)	2.1 ± 0.1	2.7 ± 0.1	2.2 ± 0.1	2.8 ± 0.1
MPT (mN)	1.9 ± 0.3	1.5 ± 0.2	1.5 ± 0.1	1.5 ± 0.1
MPS (rating 0–100)	0.1 ± 0.4	0.2 ± 0.2	0.4 ± 0.2	0.2 ± 0.2
WUR (ratio)	0.3 ± 0.3	0.4 ± 0.3	0.2 ± 0.2	0.3 ± 0.2
MDT (mN)	−0.5 ± 0.3	0.2 ± 0.5	−0.7 ± 0.1	0.2 ± 0.3
VDT (x/8)				
	Data in original units^a, b^			
	Patients		Controls	
	Tongue	Foot	Tongue	Foot
CDT (°C)	−2.39	−4.24	−0.42	−2.19
WDT (°C)	2.96	4.26	1.46	3.90
TSL (°C)	4.06	8.23	1.94	5.61
CPT (°C)	16.11 ± 5.38	13.97 ± 7.94	9.11 ± 1.60	8.35 ± 2.86
HPT (°C)	42.07 ± 2.96	42.87 ± 3.65	44.18 ± 2.14	44.95 ± 1.86
PPT (kPa)	124.5	448.6	150.0	689.33
MPT (mN)	85.62	34.30	29.3	34.08
MPS (rating 0–100)	1.26	1.47	2.65	1.73
WUR (ratio)	1.81	2.43	1.55	1.96
MDT (mN)	0.31	1.58	0.21	1.58
VDT (x/8)	6.07 ± 0.80	6.09 ± 0.86	5.68 ± 0.40	6.62 ± 0.79

^a^For CDT and WDT, differences from baseline-temperature (32 °C) are shown

^b^Retransformed mean for log-normally distributed data. All data are presented as mean ± SD. In the case of CPT, HPT and VDT, mean original data ± SD are shown. There is no evidence of occurance of DMA and PHS. In the case of CPT, HPT and VDT, mean original data are shown. All data are presented as mean ± SD. There is no evidence of occurrence of DMA and PHS

*CDT* Cold detection threshold, *WDT* Warm detection threshold, *TSL* Thermal sensory limen, *CPT* Cold pain threshold, *HPT* Heat pain threshold, *PPT* Pressure pain threshold, *MPT* Mechanical pain threshold, *MPS* Mechanical pain sensitivity, *WUR* Wind-up ratio, *MDT* Mechanical detection threshold, *VDT* Vibration detection threshold, *DMA* Dynamic mechanical allodynia, *PHS* Paradoxical heat sensation

**Table 4:** Comparison of the QST parameters in patients with burning mouth syndrome (BMS) (Results of the ANOVA – LSD posthoc analysis)

	1) Group difference			2) Area-difference			3) Interaction group/area		
	BMS versus control group			BMS versus foot					
	ANOVA Factor		LSD posthoc	ANOVA Factor		LSD posthoc	ANOVA Factor		LSD posthoc
QST Parameter	F-value	P-value		F-value	P-value		F-value	P-value	
CDT	18	<0.001 ***	<0.01 **	0.4	n.s.	n.s.	0.4	n.s.	<0.01 **
WDT	4.3	n.s.	<0.01 **	7.6	<0.05 *	<0.01 **	7.6	<0.05 *	<0.01 **
TSL	2.2	n.s.	n.s.	5.8	<0.05 *	<0.05 **	5.8	<0.05 *	n.s.
CPT	11.1	<0.01 **	<0.01 **	2.6	n.s.	n.s.	2.6	n.s.	<0.05 *
HPT	2.5	n.s.	n.s	0	n.s.	n.s.	0	n.s.	n.s.
PPT	7.8	<0.05 *	n.s.	0.2	n.s.	n.s.	0.2	n.s.	n.s.
MPT	11.3	<0.01 **	<0.001 ***	6.9	<0.05 *	<0.01 **	6.9	<0.05 *	<0.001 ***
MPS	1.8	n.s.	n.s.	2.8	n.s	n.s.	2.8	n.s.	<0.05 *
WUR	0.5	n.s.	n.s.	0	n.s.	n.s.	0	n.s.	n.s.
MDT	0.5	n.s	n.s.	4.6	<0.05 *	<0.05 *	4.6	<0.05 *	n.s.
VDT	0.8	n.s.	n.s.	2.5	n.s.	n.s.	2.5	n.s.	n.s.

1) BMS patients’ tongue compared to healthy controls’ tongue (group difference)

2) BMS patients’ tongue compared to foot in BMS patients (area difference)

3) BMS patients’ tongue compared to foot and healthy control group (interaction group/area)

QST was performed as a split study on the tongue and the foot on patients (*n* = 5) and healthy controls (*n* = 8). An ANOVA as well as a LSD posthoc (groups/ area/ interaction) was calculated to indicate main effects in the comparison of the BMS and control group, the tongue and the foot, and for the interaction among each other (interaction group/ area) *p < 0.05; **p < 0.01, ***p < 0.001; n.s. = no significance, significance shown in Fig. 2

Comparing patients’ tongue with healthy control subjects a significant difference in the perception was found for several QST parameters. In BMS patients, we measured for the tongue a deficiency in cold-perception (CDT *p* < 0.01). Regarding WDT, the difference in BMS-patients was significant (*p* < 0.01) at the tongue compared to the control group. Furthermore, we found a severe mechanical hypoesthesia at the tongue (MPT *p* < 0.001). A significant cold hyperalgesia (CPT *p* < 0.01) was detected at the tongue.

- BMS patients tongue compared to foot in BMS patients (area difference) (Tables 3 and 4, Fig. 2)

In addition, only slight differences for CDT *p* < 0.05 and PPT *p* > 0.05 were found for the patient’s feet compared to the control group. There was no statistical difference between the two groups for HPT, MPS, WUR, MDT and VDT at the tongue not the foot. No PHS occurred.

- BMS patients tongue compared to foot and healthy control group (interaction group/area) (Tables 3 and 4, Fig. 2)

Comparing patients’ foot and tongue with healthy control group LSD post hoc showed no significance.

- Contralateral uninjured side of the LNI patients compared with controls (Table 1 Fig. 1)

In one patient, we expired cold hyperalgesia on the uninjured side. Concerning other QST parameter, LNI uninjured contralateral side didn’t distinguish from healthy controls. Only for PPT, contralateral uninjured side of the LNI patient group seems to be more sensitive than the control group.

## Discussion

The original contribution of this study was to describe and objectify intraoral neurophysiological changes in patients suffering from LNI and patients with an idiopathic BMS. Therefore, we used a standardized QST protocol.

### LNI group (A)

Patients with an LNI clinically revealed a profound hypoesthesia. All of them were proved to have a reduced thermal and mechanical sensibility compared to the control group and compared to the contralateral side as well. This is in accordance with Yilmaz et al. [32] who reported a significant decreased sensitivity to cool temperature and also to warm stimuli on the injured side compared to control side. They also described a cold and heat hypoalgesia on the injured and non-injured side.

The type of injury such as local anesthetic injection, dental treatment, tumor surgery or surgery after recurrence of a peripheral giant cell granuloma did not influence testing results.

Thermal tests included detection and pain thresholds for warmth, hot and cold sensations representing C- and A-delta fiber mediated stimuli [27, 31]. All thermal tests showed significantly altered thresholds compared to control side and also to healthy individuals as an expression of a sensory deficit of the lingual nerve. According to these results a deficit of these fibers can be assumed. This was also described by Renton et al. [29], who investigated a reduced sensitivity of the injured side of the tongue to hot and cold stimuli. In their study, sensitivity on cold thresholds proved to be higher than on warm thresholds. This is in line with previous literature (Green 1984 [33] and 1987 [34]) and also confirms results of our study in which the cold detection thresholds were increased significantly compared to the contralateral side. This significantly increased threshold for detection of cold stimuli in the affected area indicates a decreased function of slightly myelinated A-delta fibers [31, 35, 36].

Jääskeläinen et al. [37] already reported long-lasting thermal hypoesthesia after unilateral nerve injury. They pointed out that thermal hypoesthesia or anesthesia recognized by the patient is characteristic for peripheral nerve injury, as seen in patients of this study.

Small, unmyelinated C-fibers were represented by the WDT and HPT. After an oral surgical treatment, WDT was shown to improve more slowly than the other tests [16]. This may proof damage to C-fibers as a negative prognostic factor [16]. Since the thermal thresholds for warm were also increased, it is to assume that damage or violation of unmyelinated C-fibers might also be involved in our patients. As a consequence, peripheral nerve damage was investigated and thermal hypoesthesia [30] and hypoalgesia could be assumed which is in line with Yilmaz et al. [32] who also proofed a cold and heat hypoalgesia in a much larger sample size.

In one LNI patient we even found cold hyperalgesia on the uninjured contralateral side as seen in the performed z-score analysis. However, further investigations showed no deviations in all other assessments and were similar to healthy controls.

These findings were in accordance with results of the mechanical tests. Mechanical pain thresholds are more suitable to detect sensory plus and minus signs [14, 16]. Affected sides of the tongue presented significantly increased PPT thresholds (C- and A-delta fiber mediated), MPT, MDT and MPS (all A-delta fiber mediated stimuli) and VDT compared to contralateral, unaffected side. VDT was significantly increased comparing the affected side of the tongue with the unaffected side, indicating damage in A-beta fibers [31, 35]. Except for PPT and VDT increased thresholds were also found comparing patients with healthy subjects. Maybe due to small sample size, there was no significant difference comparing the affected side with the volunteers for VDT and PPT. In ANOVA testing, there was no significant difference comparing both sides of the tongue, but with LSD post-hoc test LNI side compared with the volunteers showed a significant difference for WUR. This demonstrated also a deficit of A-beta fibers. This pattern of the mechanical tests showed a deficit of the affected fibers and confirmed the results of the thermal tests.

Using QST we were able to demonstrate a sensory deficit in all fiber functions, painful and non-painful stimuli for peripheral LNI. This result is confirmed by other studies [10, 30, 38, 39]. As a consequence, it is to assume that patients with LNI suffer from serious axonal injuries or separations with irreversible sensory disturbances. All of them are affected by a primary axonotmesis or neurotmesis, caused by extensive damage or destruction of axons with consecutive decrease in density of all fibers (A-beta-, A-delta-, C-fibers) which explains the sensory deficits.

### BMS group (B)

In the BMS group, CDT and WDT also showed significant deficiencies in affected patients indicating a cold/warmth-hypoesthesia and consecutively a small fiber loss. Yilmaz et al. [32] reported contrary findings. They proofed BMS patients being significantly more sensitive to cold and warm stimuli than healthy controls representing a hyperesthesia. One possible explanation might be a different patient composition which could cause a deviation of results especially in a limited patient cohort. In general, results should be interpreted with consideration of the limitations of the psychophysiological assessment tool which is highly dependant on patients’ mental abilities and compliance. Patients may also get tired and distracted. As reported in this recent study, there is also difficulty in identification of cases of heat hyperalgesia (possibly due to adaptation to warming due to the method of limits (+50 °C cutt-off point). As a possible solution, the Method of Levels was discussed to avoid tired and distracted patients and – concerning ethical guidelines – faster stimulus ramps and suprathresholds.

Evaluating profiles of neuropathic pain states, cold and warm hypoesthesia is well-known [40] and also found as a part of trigeminal nerve injury [41]. These peripheral neurologic changes coexist with other neurophysiological findings. For the tongue, a significant cold hyperalgesia was evaluated in CPT pointing to a small fiber neuropathy with central components. This cold hyperalgesia is a sign for central sensitization in patients with BMS, which was not found in patients with LNI. Yilmaz et al. [32] also documented a cold hyperalgesia compared with control patients.

A possible mechanism for a central sensitization could be found in the plasticity of neurons in the posterior horn of the spinal cord. Another explanation may be a loss of the central pain inhibition normally performed by the brain stem, the rostral ventro-medial medulla and the central gray matter. These results are also in accordance with Lauria et al. [42], who analyzed the tongues of BMS-patients by histological examination and according to Forssell et al. [30]. Both studies concluded that BMS has a neuropathic component. Primary burning mouth syndrome was also emphasized to be caused by deregulated subclinical neuropathic pain [43].

Mechanical tests (especially MPT) revealed a pinprick-hypoalgesia indicating an impaired function in small fibers. As already seen in our LNI patients, this hypofunction is a typical finding in patients suffering from peripheral nerve damage. This is in accordance with previously published results [37].

Comparing measurements of the dorsal foot in affected patients and healthy control group, all QST parameters revealed no significant differences. By using the dorsal foot as reference, a generalized neuropathy could be excluded. In this study, BMS was evaluated as a localized neuropathy with variable central and peripheral contributions among individuals as already described elsewhere [43].

Profiling patients with LNI and BMS, QST proved to be a non-invasive, psychophysical approach to profile thermal and mechanical somatosensation as already shown in previous studies [16, 27, 30, 44, 45]. Sensory signs were characterized by QST. They point to possible neurobiological mechanisms such as central or peripheral sensitization (Yekta et al., 2010b, Rolke et al., 2006a, Rolke et al., 2006b, Said-Yekta et al., 2012, Cruccu et al., 2004, Renton et al., 2006).

Because of increasing forensic implication in patients’ treatment, QST might be a useful tool to objectify clinical findings [14]. In affected patients with LNI or BMS, the monitoring of afferent nerve fiber functions is challenging and in these cases, QST might help to support decisions on further interventions. This was already emphasized by Jaaskelainen et al. [43] who described three distinct subclinical neuropathic pain states in BMS patients. They emphasized targeted treatment modalities according to these subgroups and careful neurophysiologic examinations to distinguish these groups. Also in patients with iatrogenic nerve lesions, targeted treatment modalities were presented in previous studies [9, 46]. A proper use of neurophysiological diagnostic tests seems to offer the possibility for targeted interventions based one the underlying pathophysiological mechanisms.

As an alternative to QST, sensory dysfunction in humans can be objectively quantified by electrophysiological recordings of trigeminal sensory-evoked cortical potentials [47] and brainstem reflexes [48] after stimulation of extraoral and intraoral sites [44, 49]. Functional magnetic resonance imaging may also reveal sensory functions [50, 51]. But these methods are complex, and still more time consuming than QST and do not allow the identification of isolated sensory deficiencies and therefore seem not to be appropriate in clinical routine. The QST protocol used in this study remains time consuming in clinical routine diagnostic. Some studies [29, 52] restricted screening to thermal QST to examine the tongue. By extending the protocol to include mechanical components however, a differentiated classification of nerve fiber function is possible. Previous studies [6, 53] emphasized to reduce the QST battery to only seven parameters. In order to benefit from the entire potential of QST based diagnosis, exclusion of parameters can’t be recommended because essential information concerning hypo- and hyperesthesia would be missing.

## Conclusions

The present study used the standardized QST battery in patients with LNI and BMS. Different nerve fiber functions in nine patients with sensory disturbances were evaluated and compared with healthy controls. Thermal and mechanical tests allowed an accurate topographical diagnosis of nerve lesions and a determination of the profile, type and severity of LNI and BMS patients. QST seems to offer a valid option to profile somatosensory nerve fiber function and can be applied for non-invasive assessment of sensory nerve function.

The results indicate that patients with LNI suffer from a peripheral neuropathy. The increased thermal and mechanical thresholds point to a serious axonal injury or separation and recovery seems to be doubtful. BMS could be seen as neuropathy with variable central and peripheral contributions among individuals resulting in chronic pain. Targeted therapy options may follow after neurophysiological diagnosis. This protocol proved to be suitable to identify and precisely describe peripheral and central nerve fiber function in the orofacial area.

## Abbrevations

BMS: Burning mouth syndrome
CDT: Cold detection threshold
CPT: Cold pain threshold
DFNS: German Research Network on Neuropathic Pain
DMA: Dynamic mechanical allodynia
HPT: Heat pain threshold
IANI: Inferior alveolar nerve injury
LNI: Lingual nerve impairment
LSD: Least significant difference
MDT: Mechanical detection threshold
MPS: Mechanical pain sensitivity
MPT: Mechanical pain threshold
PHS: Paradoxical heat sensation
PPT: Pressure pain threshold
QST: Quantitative Sensory Testing
SD: Standard deviation
TSL: Thermal sensory limen
VDT: Vibration detection threshold
WDT: Warm detection threshold
WUR: Wind up ratio
